# Effects of Environment and Sowing Time on Growth and Yield of Upland Cotton (*Gossypium hirsutum* L.) Cultivars in Sicily (Italy)

**DOI:** 10.3390/plants9091209

**Published:** 2020-09-15

**Authors:** Teresa Tuttolomondo, Giuseppe Virga, Francesco Rossini, Umberto Anastasi, Mario Licata, Fabio Gresta, Salvatore La Bella, Carmelo Santonoceto

**Affiliations:** 1 Department of Agricultural, Food and Forest Sciences, Università degli Studi di Palermo, 90128 Palermo, Italy; teresa.tuttolomondo@unipa.it; 2 Research Consortium for the Development of Innovative Agro-environmental Systems (CoRiSSIA), Via della Libertà 203, 90143 Palermo, Italy; giuseppe.virga@corissia.it; 3 Department of Agricultural and Forest Sciences, Università degli Studi della Tuscia, 01100 Viterbo, Italy; rossini@unitus.it; 4 Department of Agriculture, Food and Environment, Università degli Studi di Catania, 95123 Catania, Italy; umberto.anastasi@unict.it; 5 Department of Veterinary Sciences, Università degli Studi di Messina, 98168 Messina, Italy; fabio.gresta@unime.it; 6 Department of Agraria, Università degli Studi Mediterranea di Reggio Calabria, 89122 Reggio Calabria, Italy; csantonoceto@unirc.it

**Keywords:** *Gossypium hirsutum*, environment, sowing times, seed and lint yields, indices of agronomic earliness, Sicily

## Abstract

Cotton is one of the most important industrial crops in the world. Though widely cultivated in Sicily (Italy) in the past, cotton growth on the island has disappeared today due to a complex variety of agronomic, economic and socio-political reasons. In recent years, increased interest in natural fibers worldwide has led to a revival in cotton plants in the Mediterranean area. The aim of this paper was to assess the response of *Gossypium hirsutum* L. cultivars to different environments and sowing times. Elsa and Juncal were selected from the most promising cotton cultivars regarding earliness and productivity. Plants were tested with three sowing times and in two Sicilian environments. Cotton yield and yield components were significantly affected by experimental station, sowing time and cultivar. Lint yield of cultivars was 1.60 t ha^−1^ on average, and the highest value of 1.99 t ha^−1^ was obtained from an early sowing time. The three indices of agronomic earliness varied significantly based on treatments. In conclusion, the evaluation of response genotype-by-environment under different sowing times could represent a strategy to obtain optimal cotton seed and lint yields, although other general aspects, such as labor costs, land availability and capital resources, should be also considered when evaluating the reintroduction of the species in Sicily.

## 1. Introduction

Upland cotton (*Gossypium hirsutum* L.) is an annual open field crop belonging to the *Malvaceae* family. It is a semi-xerophytic species native to North and Central America, and Mexico and grows well in tropical and subtropical climate conditions. Although it shows marked adaptation ability to a large variety of soils and, of particular interest, it is semi-tolerant to salinity [1], it is not cultivated in all geographic areas of the world [2] and it shows sensitivity to water logging [3,4,5]. 

From an economic point of view, it is one of the most important industrial crops in the world [6], cultivated for the production of both fiber and oilseed. Cotton fibers contain 90–95% cellulose, in addition to waxes, pectins, organic acids and inorganic substances. The fibers demonstrate excellent physical properties, making this species the main source of natural fiber in the world [7,8,9]. Upland cotton is also a source of relatively high-quality proteins and one of the best oil-producing crops in the world [10,11,12], as the seed is exceedingly rich in oil and proteins.

However, the literature highlights that fiber and seed yields can vary significantly based on climate conditions [13,14,15,16,17] and agronomic practices such as plant density, sowing time, irrigation and fertilization [18,19,20,21,22,23,24,25,26]. In particular, a number of authors maintain that sowing time should be considered one of the most important agronomic factors affecting not only growth and yield components of the species but also fiber quality [20,21,27,28,29].

A suitable sowing time is fundamental for root penetration and vegetative growth in order to ensure optimal uptake of available soil nutrients and solar energy [21]. However, choosing the best sowing time may not be easy due to the effects on crop production of environmental factors, such as air and soil temperatures, and solar radiation. All of these factors can play a major role in deciding the best sowing period and can affect the length of critical phenological stages of the species [30,31,32,33]. 

As stated by some authors [27,34], early planted cotton can exploit the rainfall, temperature and sunlight levels that occur in spring and summer, and start growth and reproduction stages earlier, thereby producing more blooms and setting a greater number of bolls. However, sowing too early can determine poor stand establishment, limited plant growth and increased susceptibility of the seedling to disease due to lower air and soil temperatures, and pest incidence [35,36]. In contrast, late planting can significantly reduce lint yield and induce low boll weight due to delayed physiological maturity and carbohydrate deficiency [20,37,38,39]. 

In Italy, the cultivation of upland cotton has slowly disappeared over the years and, today, there are no data on cultivation of the species in the country from official sources of Italian statistics [40]. This decline in cotton cultivation can be explained by various agronomic, economic and social factors, such as the low cash value of its production, lower yields obtained in non-irrigated conditions, the lack of farm workers in this area and increasing labor costs. 

Upland cotton was the subject of intense study in the 1980s as part of the “Cotone” project financed by the Italian Ministry of Agriculture and Forestry, which included a series of test trials in southern Italy. A number of cotton cultivars were compared and experimental tests were carried out to collect information on the effects of cultivation practices on plant growth and yield [41,42,43,44,45]. The species was found to be highly adaptable to various environments, but marked differences in lint and seed yields were obtained from the effects of environmental, technical and varietal factors. Furthermore, a number of tests highlighted the fact that morphological, physiological and production aspects of cultivars and the choice of suitable sowing dates needed to be given priority in order to improve and increase cotton boll yields. 

Current signs would seem to suggest that the cotton plant should be reassessed and given due consideration for reintroduction in southern Italy, particularly considering the deficit in supply in Italy, and in Europe in general, and the fact that it is increasingly difficult to find suitable crops to broaden crop ranges. However, despite the availability of high-yielding cotton cultivars, the reintroduction of the species into these areas should also take other aspects of cultivation, such as land availability, labor costs and capital resources into consideration. In Sicily (Italy), the main problems linked to cotton cultivation (lower yields and high production costs) could be solved with irrigation and the mechanization of a number of cropping practices; however, it is essential to update agronomic techniques and introduce more productive cultivars. 

This paper reports the results of two-year trials on upland cotton cultivars within the research project “Assessment of morphological, biological and agronomic characteristics of cotton cultivars for the re-introduction of this species into Sicily”. The main aim of this paper was to assess the response of two upland cotton (*Gossypium hirsutum* L.) cultivars in two Sicilian environments and to three sowing times, after a preliminary screening of the 15 cotton cultivars.

## 2. Materials and Methods

### 2.1. Experimental Sites, Climatic Data and Cultivars

Field studies were conducted in 2011 and 2012 at the “Piana di Gela contrada Rinazzi” (ExpSt_1) experimental station (Gela, 19 m a.s.l., 37°05′25″ N, 14°16′55″ E) belonging to the University of Reggio Calabria, south-east Sicily and at the “Orleans” experimental farm (ExpSt_2) (Palermo, 31 m a.s.l., 38°06′26.2″ N, 13°20′56.0″ E) at the University of Palermo, northwest Sicily. The photos of the two experimental fields are presented in Supplementary Figures S1 and S2.

#### 2.1.1. “Piana di Gela Contrada Rinazzi” Experimental Station

Soil at ExpSt_1 area was clay loam (23% sand, 45% clay, 32% silt) with a pH 7.30, 174 g kg^−1^ total carbonates, 74 g kg^−1^ active carbonates, 0.80 g kg^−1^ total nitrogen, 8.00 g kg^−1^ organic carbon, 34 mg kg^−1^ assimilable phosphorus and 328 mg kg^−1^ exchangeable potassium. According to the Köppen–Geiger climate classification [46], the study location is characterized by a warm temperate Mediterranean climate with dry, hot summers. Annual average rainfall is approx. 400 mm, mainly distributed between October and April. With reference to time series 1970–2010, the annual average temperature was 17.4 °C, annual average maximum temperature was 20.7 °C and annual average minimum temperature was 14.5 °C. 

#### 2.1.2. “Orleans” Experimental Station

Soil at ExpF_2 area was sandy clay loam (56% sand, 23% clay, 21% silt) with a pH of 7.91, 19 g kg^−1^ organic carbon, 58 g kg^−1^ total carbonates, 37 g kg^−1^ active carbonates, 13.2 g kg^−1^ total nitrogen, 18.11 mg kg^−1^ assimilable phosphorus and 320 mg kg^−1^ exchangeable potassium. The climate of the area is Mediterranean with mild, humid winters and hot, dry summers [46]. The annual average temperature is 18.4 °C, with annual average minimum and maximum temperatures of 14.8 and 21.7 °C, respectively. Annual average rainfall is approx. 600 mm. 

#### 2.1.3. Climatic Data

Data on rainfall and air temperatures were recorded by two weather stations, one for each field, belonging to the Sicilian Agro-Meteorological Information Service [47] located near the experimental fields. Each station was synchronized with Greenwich Mean Time (GMT) in order to operate using synoptic forecast models. The stations were equipped with an MTX datalogger and various sensors for the measurement of air temperature, solar radiation, leaf wetness, relative humidity, total rainfall and wind speed. 

#### 2.1.4. Cultivars

In 2011, 15 cotton cultivars (14 *Gossypium hirsutum* L. and 1 hybrid *Gossypium barbadense* L.) were compared at ExpSt_1, only (Table 1). 

**Figure 1:**
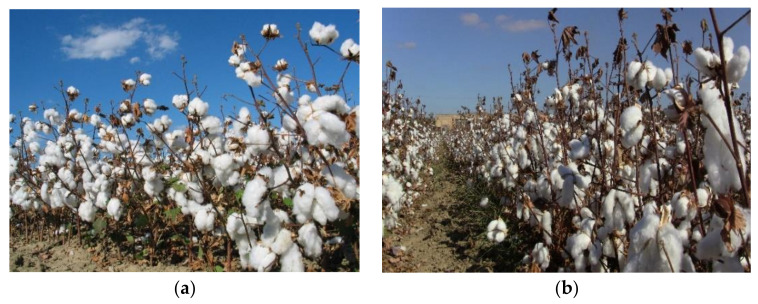
The two cultivars of *Gossypium hirsutum* L. used in second year trials: (**a**) Elsa; (**b**) Juncal.

In 2012, Elsa and Juncal (Figure 1), belonging to *Gossypium hirsutum* L., were selected from those cotton cultivars which obtained the best results in terms of earliness and productivity in 2011. However, appreciable results were also found in other cultivars, such as 5a, belonging to *Gossypium barbadense* L. Although the different agronomic behavior of *Gossypium hirsutum* and *Gossypium barbadense* in terms of yield and fiber characteristics, in this study, having to assess the most appropriate sowing time, it was decided to grow two cultivars of upland cotton in two experimental stations in order to investigate the effects of different environments and sowing times on morphological and yield characteristics.

### 2.2. Experimental Design and Main Cultivation Practices

#### 2.2.1. Agronomic Management during 2011

In 2011, at ExpSt_1, a complete randomized block design was used with three replications containing the 15 cotton cultivars. Each plot measured 25 m^2^ (5 m × 5 m). Soil was ploughed to a depth of 35 cm, tilled and subsequently disinfested with chlorpyrifos at a rate of 0.225 kg ha^−1^. The previous crop was artichoke. Before sowing, 110 kg ha^−1^ N, 110 kg ha^−1^ P_2_O_5_ and 80 kg ha^−1^ K_2_O were distributed. Two fertigations of 10 kg ha^−1^ N each were also applied. All cotton cultivars were sown on 9th May. Sowing was carried out mechanically using a pneumatic seeder with rows spaced 1 m apart. Plant density was 12 plants m^2^. Two treatments with plant growth regulator 1, 1 dimethylpiperidine chloride, at a rate of 0.50 l ha^−1^ (19 g ha^−1^), were administered. At the pre-emergence stage, graminaceous and dicotyledonous weeds were controlled chemically using pendimethalin 31.7% and s-metolachlor 87.3%. At the post-emergence stage, weeds control was carried out mechanically. Regarding insect control, the cotton aphid (*Aphis gossypii* Glover), the cotton bollworm (*Helicoverpa armigera* Hübner) and the smaller green leafhopper (*Empoasca vitis* Göethe) were managed with insecticides imidacloprid, indoxacarb and thiamethoxam, respectively. Drip irrigation was carried out four times during the growth stages of cotton and 50 mm of water was applied during each irrigation. Cotton bolls were manually harvested on 16th and 25th September and on 1st and 7th October. 

#### 2.2.2. Agronomic Management during 2012

In 2012, a split-split-plot design was used with three replications. The main plot was experimental station, the sub-plot was sowing time and the sub-sub-plot was cultivar. Tests were conducted on plots of different size; each plot measured 23.75 m^2^ (5 m × 4.75 m) at ExpSt_1 and 15.20 m^2^ (4 × 3.80 m) at ExpSt_2. The soil was ploughed to a 30 cm depth, tilled and disinfested with chlorpyriphos at a rate of 0.225 kg ha^−1^. Before sowing, both experimental stations received a total of 110 kg ha^−1^ N, 110 kg ha^−1^ P_2_O_5_ and 80 kg ha^−1^ K_2_O. At post-emergence stage, three N fertilizations of 30 kg ha^−1^ each were applied during various growth stages of upland cotton. The two cultivars Elsa and Juncal were sown on 31st March, 21st April and 12th May at ExpSt_1 and on 19th April, 30th April and 14th May at ExpSt_2. Sowing was carried out manually with rows spaced 0.95 m apart. Plant density was 25 plants m^−2^ at both experimental stations. A single treatment of plant growth regulator 1, 1 dimethylpiperidine chloride at a rate of 0.50 l ha^−1^ (19 g ha^−1^), was administered one month after each sowing event. Weed control was carried out using pendimethalin 31.7% and s-metolachlor 87.3% at pre-emergence stage and mechanical weeding at post-emergence stage. Indoxacarb was applied to prevent cotton bollworm attacks on plants. The 4 (ExpSt_1) and 6 (ExpSt_2) drip irrigation events were effectuated during the growth stages of the cotton, supplying 50 mm (ExpSt_1) and 35 mm (ExpSt_2) of water during each irrigation event. Cotton bolls were manually harvested on 7th, 14th, 22nd and 29th September at ExpSt_1 and on 22nd and 29th September and 6th and 13th October at ExpSt_2. Harvesting was carried out at different times in order to calculate the indices of agronomic earliness.

### 2.3. Agronomic Parameters

The main growth stages of cultivars were determined according to extended BBCH-scale [48]: emergence (70% of plants with cotyledons completely unfolded), beginning of flowering (50% of plants with at least one open flower) and beginning of boll opening (50% of plants with at least one open boll). Growing degree days (GDDs) were used to describe crop phenology. Subsequently, daily GDDs were calculated for each phenological stage with the Equation (1):

(1) $$\mathrm{GDD}=\frac{\left(T_{\max}+T_{\min}\right)}{2}-T_{b}$$

where: T_max_ and T_min_ are daily maximum and minimum air temperatures and T_b_ is the base temperature below which development ceases. A value of 15.6 °C was used as the base temperature for the cotton plant [49,50]. Accumulated GDDs for each phenological stage were calculated by summing daily GDDs at each stage [27].

At each harvest, the number (no.) of bolls per plant and average boll weight (after oven drying) were determined. At the end of the final harvest, plant height, height of first fruiting branch and number of open bolls per plant were measured from a pool of 25 (ExpSt_1) and 10 plants (ExpSt_2), randomly selected from each plot. Finally, cotton lint was separated from seeds of raw material harvested from the plot, using a lab saw cotton gin machine (Supplementary Video S1). Lint and seed yields, and lint and seed percentages were subsequently determined.

Three main indices of agronomic earliness were also calculated: mean maturity date (MMD), production rate index (PRI) and earliness index (EI). Mean maturity date [51,52] was used to determine the timing of crop maturation in cotton cultivars; the quicker the maturation time, the greater the uniformity and the lower the risk to crops from decreasing air temperatures and rainfall levels that could occur in the final stages of the crop cycle. It was calculated with the Equation (2):

(2) $$MMD=\frac{\left(W_{1}\times H_{1})+(W_{2}\times H_{2})+(W_{n}\times H_{n}\right)}{W_{1}+W_{2}+W_{n}}$$

where: W = lint weight; H = number of days from sowing to harvest; 1, 2, *n* = consecutive periodic harvest number. 

The production rate index [52,53] was used to assess both the earliness and productivity of cotton cultivars. It was calculated with the Equation (3):

(3) $$PRI=\frac{W_{1}+W_{2}+W_{n}}{MMD}$$

The earliness index [54] was used to measure the earliness of the cultivars as a percentage ratio of the lint weight related to the first harvest and the total production obtained from all harvests. It was calculated with Equation (4):

(4) $$EI=\frac{W_{1}}{W_{1}+W_{2}+W_{n}}\times 100$$

### 2.4. Statistical Analyses

Statistical analyses were performed using the packages DSAASTAT version 1.1 and MINITAB 19 for Windows. Data were compared using analysis of variance. The difference between means was carried out using the Tukey test.

## 3. Results

### 3.1. Rainfall and Temperature Trends in the Experimental Stations

#### 3.1.1. Analysis of Rainfall and Temperatures during the 2011

**Figure 2:**
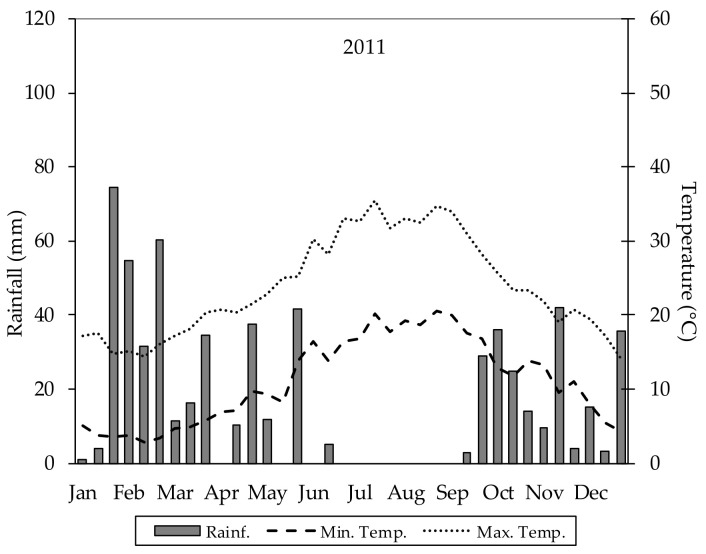
Rainfall and temperature trends in 2011. Graph refers to ExpSt_1 area.

In ExpSt_1 (when considering growth stages of cotton cultivars) from May to October, the air temperature was 13.6 °C on average. Temperatures increased from May to the second 10-day period in July. The highest minimum and maximum temperatures were 20.21 and 35.49 °C, respectively. In particular, from late June to mid-September, maximum air temperatures were constantly above 30.00 °C. Accumulated GDDs from May to October were 1312.67. Rainfall events (59.61 mm), which occurred before sowing time (from April to early May), allowed an increase in the soil water reserve; however, they also caused a delay to sowing time, which was scheduled originally for the third 10-day period in April. Regarding other rainfall, only that which fell in May (a little over 40 mm) was useful for plant growth. A total lack of rainfall was recorded between June and the first 10-day period in September. In the final stages of the crop cycle, rainfall was recorded again (Figure 2).

#### 3.1.2. Analysis of Rainfall and Temperatures during 2012

In both the experimental stations, average air temperature and rainfall trends were consistent with multi-year averages (Supplementary Tables S1 and S2).

Temperatures increased steadily from April to August at both experimental stations. In September, maximum and minimum temperatures decreased greatly. The highest and lowest average air temperatures were recorded at ExpSt_1. In particular, the highest maximum temperature (36.19 °C) was recorded during the second 10-day period in July, and the lowest minimum temperature (6.45 °C) was recorded during the second 10-day period in April. When comparing air temperatures between April and September, we observed that minimum temperatures were on average 4.76 °C lower and maximum temperatures were 1.95 °C higher at ExpSt_1 than at ExpSt_2. In April and May, when the three sowing times were carried out, average minimum temperatures were lower at ExpSt_1 by 4.89 °C (April) and 5.13 °C (May) compared to ExpSt_2. 

Accumulated GDDs were 1459 (ExpSt_1) and 1581 (ExpSt_2) from the first 10-day period in April to the first 10-day period in October. During the crop cycle, total rainfall was different in the two areas. It was higher at ExpSt_2 (153 mm) than at ExpSt_1 (119 mm). The highest rainfall levels (48.60 mm) occurred during the second 10-day period in April at ExpSt_1. 

**Figure 3:**
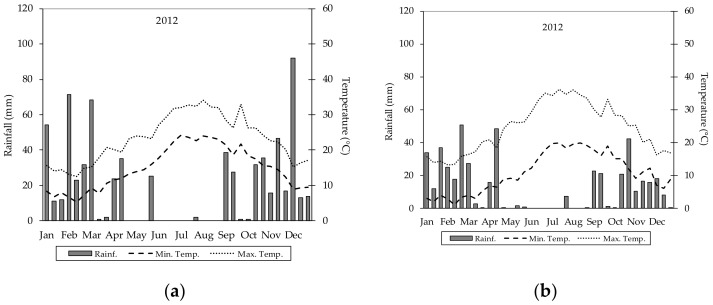
Rainfall and temperature trends in 2012. Graph (**a**) refers to ExpSt_1 area; graph (**b**) refers to ExpSt_2 area.

Rainfall distribution was not uniform throughout the growth stages of the plants, and rainy days were more concentrated in the spring than in the summer. It is worth noting that, in both experimental stations, the most significant rainfall events occurred during the first two 10-day periods in April and September. Rainfall occurring in April was useful for plant growth and production in particular (Figure 3).

### 3.2. Agronomic Assessment of 15 Cotton Cultivars during the 2011 

Results of one-way ANOVA revealed significant differences between the 15 cotton cultivars in the study for all agronomic and phenological stages tested (Table 2). 

A density of 11.2 plants m^−2^ was obtained on average for the cultivars; this was similar to the planned density of 12 plants m^−2^. The beginning of flowering occurred, on average, within 77 days from sowing. With the exception of 5 A (hybrid), which showed the shortest time interval (69 days from sowing) between the two stages, few differences were found between the cotton cultivars in terms of this specific time interval “sowing to flowering”. The interval ranged from 72 days (Juncal) to 80 days (Flora and Julia) from sowing. Juncal differed from those cultivars which began flowering stage 76 days from sowing. Regarding beginning of boll opening, the cultivars showed even fewer differences in terms of time interval from sowing stage. Beginning of boll opening occurred, on average, within 121 days from sowing. 1J, 2D and Alexandro (118 days) recorded the earliest boll opening time, while ST 373, ST 457 (123.40 days) and DP 419 (124 days) the latest. 

Cotton cultivars were significantly different as regards the three indices of agronomic earliness in the study. The mean maturity days index was 136.70 days on average and ranged from 138.60 days (Claudia) to 134.60 days (Alexandro and Juncal). Alexandro, Juncal and 3 C differed from those cultivars showing an average ripening time of seed above 137.10 days. The production rate index was 19.50 kg ha^−1^ day^−1^ and showed significant differences between the cultivars, similar to those recorded for lint yield. This index ranged from 23.30 kg ha^−1^ d^−1^ for Elsa to 15.80 kg ha^−1^ d^−1^ for 3 C. It is worth noting that Elsa differed significantly from those cultivars which obtained daily lint production below 19.80 kg ha^−1^ d^−1^. The Earliness Index was 39.90% on average. 

In particular, Alexandro (54.10%) and Juncal (53.20%) recorded the highest values of this index and differed significantly from those cultivars which obtained less than 40% of lint at the time of the same harvest.

Furthermore, analysis of variance showed that cotton cultivars were also significantly different for all the morphological and productive traits in the study (Table 3). 

Plant height ranged from 57.91 cm (Flora) to 83.50 cm (5 A), with an average height of 68.70 cm. Cotton cultivars showed significant differences regarding height of first fruiting branch on the stem per plant. Greater variability was observed between replications regarding this morphological trait in particular. Maximum and minimum heights of first fruiting branch per plant were 23.30 (Elsa) and 14.20 (Alexandro) respectively, with an average height of 18.60 cm. 

Significant differences between cultivars were recorded for number of open bolls per plant. The highest number of open bolls per plant was found in 5 A (6.80) while the lowest numbers on average were found in 3 C (4.50), Claudia (4.60), Flora and Julia (4.80). The average number of open bolls per plant was 5.40. Maximum boll weight (5.12 g) was recorded in Celia, while cultivar 5 A produced the minimum weight (3.84 g, on average). Average boll weight was 4.48 g. 

As regards other yield traits, significant differences were found for lint yield (1.61 t ha^−1^ on average). The highest yields (range 1.52–1.84 t ha^−1^) were recorded for Elsa, Alexandro, 5 A, DP 419, Juncal, 2 D, ST 273 and 1 J, while the lowest yields (range 1.35–1.57 t ha^−1^) for Claudia, 4 T, Flora, Celia, ST 457, Julia and 3 C. Average cotton seed and lint percentages were 60.50% and 39.50%, respectively. In particular, cotton lint ranged from 32.70% (5 A) to 44.90% (Claudia). 

### 3.3. Cotton Plant Phenology and Earliness during 2012

The length of the main phenological stages of the cotton cultivars and the relative accumulated GDDs, relating to each sowing time and to each experimental station, are shown in Table 4. 

At ExpSt_1, the number of calendar days from sowing to start emergence, beginning of flowering and beginning of boll opening was greater for the early-sown crop (31th March) compared to the normal (21th April) and the late-sown crop (12th May). When considering accumulated GDDs at each phenological stage, the early-sown crop showed the fewest GDDs to start the main phenological stages compared to the late-sown crop. In contrast, the number of accumulated GDDs required by the normal sowing time to start each phenological stage was similar to that of early sowing time. 

At ExpSt_2, a slightly different trend was observed as regards both the number of calendar days and accumulated GDDs required in order to begin the various phenological stages. In fact, the cotton plants required a similar number of calendar days to start both emergence and beginning of flowering, regardless of sowing time. It was also found that late-sown plants (14th May) required the fewest calendar days to start beginning of boll opening compared to early (19th April) and normal (30th April) sown plants. It was also observed that late sowing times required the fewest GDDs to start emergence and beginning of boll opening compared to other sowing times, whereas early sowing times required the fewest GDDs to start beginning of flowering.

With regard to the influence of the main factors on phenological stages, it was found that experimental station and sowing time had a significant effect on cotton plant phenology, but that cultivar was non-significant for all the stages in the study (Table 5). Results of analysis of variance revealed, however, that the interactions between the main factors were non-significant for all the phenological stages. 

The number of days between sowing and emergence were greater at ExpSt_1 (15 days, on average) than ExpSt_2 due to the fact that sowing occurred at ExpSt_1 many days earlier (when air and soil temperatures were less favorable for seedling emergence) than at ExpSt_2 (data of soil temperature were not shown). The longest period between sowing and emergence, however, was observed with the early sowing times. Data from late sowing times show a significant increase in the number of days between sowing and emergence compared to normal sowing times, undoubtedly due to lower air temperatures, above all at ExpSt_1. Beginning of flowering (Supplementary Figure S3) occurred 3 days earlier at ExpSt_2 than ExpSt_1 due to better climate conditions, thus influencing the duration of the subsequent growing stage. 

Significant differences between early and late sowing times were found regarding the period between sowing and beginning of flowering, which ranged from 86 days (early sowing time) to 76 days (late sowing time). 

The high significance found for the interaction experimental station-by-sowing time highlights, on the other hand, that, for each of the three sowing times, differences in the sowing-beginning of flowering period were consistent with the effects of the main two factors. The same relationship was observed for the experimental station-by-cultivar interaction. With regard to sowing-beginning of boll opening (Supplementary Figure S4), this period was found to be longest at ExpSt_2 due to better agronomic conditions. The number of days between sowing-beginning of boll opening decreased from 145 days (early sowing time) to 123 days (late sowing time).

The experimental station-by-sowing time interaction showed significant differences, which were consistent with the effects of the two factors. In particular, the shortest period of sowing-beginning boll opening was found for the normal sowing time at ExpSt_1 (124 days) and the late sowing time at ExpSt_2 (128 days). It is worth noting that the interaction between the three main factors in the study also determined significant differences for this inter-stage period and that the beginning of boll opening occurred earlier at ExpSt_2 than ExpSt_1 for Elsa and Juncal during the third sowing time (Supplementary Table S3). 

The indices of agronomic earliness for the cotton were often significantly affected by the three main factors, although not always (Table 5). 

The interactions between the main factors produced significant differences for all the indices of agronomic earliness except, those regarding experimental station-by-sowing time and experimental station-by-sowing time-by-cultivar for PRI. MMD varied significantly between the two experimental stations. Average seed ripening time for the two cultivars was longest (166 days) at ExpSt_1 compared to ExpSt_2. Differences found between the three sowing times were less pronounced than the differences between the two experimental stations. At S_3_, a higher MMD value of 161 days was recorded compared to earlier sowing times. No significant differences were observed for the two cultivars. The main interactions of the three factors determined significant effects for MMD; in particular, the interaction effect experimental station-by-sowing time-by-cultivar highlights the fact that, at ExpSt_2, Elsa obtained the lowest values of MMD at S_2_ and Juncal at S_2_ and S_3_. PRI varied significantly with sowing times, with average values ranging between 17.90 kg ha^−1^ d^−1^ (S_3_) and 29.84 kg ha^−1^ d^−1^ (S_1_). Elsa achieved a higher average value (25.41 kg ha^−1^ d^−1^) of PRI compared to Juncal. The experimental station-by-cultivar and sowing time-by-cultivar interactions determined significant effects on PRI only. The percentage ratio between the lint weight related to the first harvest and the total production obtained from all harvests was significantly lower in ExpSt_1 than ExpSt_2. When considering the sowing time, EI showed the highest value at S_2_. Elsa achieved the highest average value (59.38%) of EI. All interactions between the main factors had significant effects on EI. The experimental station-by-sowing time-by-cultivar interaction effect highlights the fact that, at ExpSt_1, Juncal obtained the lowest value of EI at S_3_ compared to other combinations. However, this was not observed at ExpSt_2 (Supplementary Table S3).

### 3.4. Cotton Plant Growth Characteristics, Yield and Yield Components during 2012

Experimental station had a significant effect on plant height and height of first fruiting branch per plant, but no significant effect of sowing time and cultivar were observed. Yield and yield components were, in contrast, significantly affected by experimental station, sowing time and cultivar, except regarding the number of open bolls per plant. Results of analysis of variance revealed that the interactions between the main factors were non-significant for all the growth characteristics and yield traits (Table 6). 

Plant height ranged from 87.93 cm (ExpSt_1) to 103.10 cm (ExpSt_2), on average. Despite the fact that height represents a morphological and physiological trait, it was not significantly affected by sowing time. However, greater variability in the height of the first fruiting branch per plant was observed between experimental stations. The highest value (67.42 cm) of this trait was recorded at ExpSt_2. The number of bolls per plant was not influenced by the main factors. In contrast, boll weight varied only with experimental station, with average values ranging between 3.88 g (ExpSt_1) and 5.03 g (ExpSt_2). 

Lint yield was significantly affected by experimental station, sowing time and cultivar, and was found to be 1.60 t ha^−1^ on average for the two cultivars. The highest lint yield of 1.72 t ha^−1^ was produced by Elsa. Seed yield was significantly affected by experimental station and sowing time only. The highest seed yield (2.39 t ha^−1^) was obtained at ExpSt_1. The maximum ranges for yield and yield components, for the three factors in the study, were recorded for the sowing time factor. In particular, early sowing time produced the highest lint and seed yields, progressively decreasing to the late sowing time. The highest lint percentage (42.17%) was recorded at ExpSt_1, and Elsa obtained the highest performance (42.80%). The effect of sowing time on lint percentage was not significant.

With regard to interaction effects, significant differences were found for the height of the first fruiting branch per plant. No significant effects of the experimental station-by-cultivar and sowing time-by-cultivar interactions were recorded for plant height. It is interesting to observe, however, that the interaction between the three main factors had a significant effect both on plant height and height of first fruiting branch per plant. All the traits of crop production, except seed yield, were significantly affected by the experimental station-by-sowing time. However, experimental station-by-cultivar interaction determined significant differences for both lint and seed yields. At ExpSt_1, a higher lint percentage (42.50%) was recorded at the early sowing time compared to successive sowing times (Supplementary Table S3). 

Finally, it is worth noting that, in both experimental stations, the accumulation of GDD was different for each sowing time (Table 7). 

In particular, when considering the main development stages from sowing to harvest, early sowing time had the highest GDDs, while late sowing time had the lowest. The relationship between the accumulated GDDs and the average lint and seed yield values for each sowing time in both experimental stations confirms that the early dates of 31st March for ExpSt_1 and 19th April for ExpSt_2 represent the best sowing times to obtain higher cotton yields. 

## 4. Discussion

In this study, in first year trials, 15 cotton cultivars of Greek and Spanish origin were assessed in an area where horticultural crops, such as artichoke, are traditionally cultivated. All the cultivars adapted well to the semi-arid climate conditions of the study area and showed significant differences for the main phenological, morphological and yield traits. These differences were probably due to genetic and environmental factors, as stated by the literature. Some authors [55,56] have, in fact, reported that genetic variation can significantly affect seed and lint yields of this crop and its components. Others [57,58,59] have noted that environmental factors, such as air temperature, rainfall and wind can significantly influence yield components and the qualitative characteristics of the cotton fiber.

The main differences between the cultivars in terms of yield components were due to genotype response to environmental conditions. In particular, lint yield ranged from 1.61 to 1.83 t ha^−1^ on average for a group of eight cultivars. The literature reports values ranging between 1.00 and 2.00 t ha^−1^ on average, mainly due to genetic traits of cultivars and cultivation practices, such as planting density, sowing time and nutrient fertility management [18,20,21,59,60]. The cotton seed and lint percentage values of the 15 cotton cultivars were similar to those reported in the literature [18,19,20,21,22,60,61]. 

It is worth noting that the findings in this study were compared to those obtained in other countries in the world where climate and soil conditions are markedly different. No up-to-date information is available on cotton lint yield and component values in Italy due to the disappearance of the species from Italian cropping systems. In Sicily, in an often-cited 1993 study [62], which was carried out in the same study area (ExpSt_1), boll weight of cotton cultivars was 5.31 g, lint yield was 1.60 t ha^−1^, and lint percentage was 39%, on average. Comparing these yield values to those in our study, it was shown that yield component values were on average very similar. This can be explained considering both the environmental conditions and main cultivation practices, which were not substantially different from those in our study. 

However, despite the fact that seed and lint yields allow us to assess the agronomic performance of a set of cultivars, earliness also represents a selective intrinsic characteristic of the species. This must also be considered highly relevant as earliness can improve the ability of this crop to carry out its growth cycle from mid-April to end of September in the Mediterranean, for example. In our study, low variability between the cultivars in terms of beginning of flowering, beginning of boll opening (biological earliness) and MMD (agronomic earliness) would explain the fact that early genotypes tend to be preferred in Greece and Spain as they help overcome the limits of the Mediterranean climate in the initial and final stages of their growth cycle. In 2012, a series of positive conditions for the growth and yield of the species occurred, such as favorable air temperatures from emergence stage to complete boll opening and the absence of insect attacks. It is worth noting that, in both the experimental areas, average air temperature and rainfall trends were consistent with multi-year averages, as the variation in climatic factors was very low over the years. Our findings highlighted the fact that the experimental station had a significant effect on cotton plant phenology, and this was in agreement with previous studies [59,63] which showed that air temperature and rainfall can influence the length of the various crop growth stages. Sowing time also determined significant differences regarding the length of the periods between sowing and emergence, sowing and beginning of flowering, and sowing and beginning of boll opening. In particular, fewer days between sowing and first boll opening induced by a late sowing time can be explained by the fact that late-sown crops developed quickly with the higher air temperatures, leading to a reduction in vegetative growth stages and a shorter reproductive period [21,64]. 

With regard to plant height and the height of the first fruiting branch, significant differences between the two experimental stations can be explained by the differing minimum and maximum air temperatures in the two areas during cotton growth stages, in addition to a number of cultivation aspects, such as auxiliary water requirements of the species. In particular, higher maximum air temperatures, rainfall scarcity during the spring and summer months and lower field capacity at ExpSt_2 determined an increase in the number of irrigation events, which promoted plant growth. 

Concerning cotton yields, the choice of sowing time significantly affected lint yields, seed yields, and yield components. In fact, the two cultivars obtained the highest lint and seed yields at early sowing time; Elsa performing better than Juncal in terms of lint yield. Accumulation of higher GDDs for early sowing times at both experimental stations highlighted the fact that the early dates represented the best sowing dates for cotton crops in order to obtain higher lint and seed yields in Sicilian areas. A greater accumulation of GDDs determined an increase in yield and yield components due to the fact that air temperature is considered the climate factor most able to influence and govern plant growth rate and yields [65,66]. On the contrary, at both experimental stations, late sowing was not considered optimal as it resulted in a reduction in lint and seed yields due to the fact that the plants completed their cycle more quickly and accumulated fewer GDDs. 

Our findings were consistent with previous studies [20,21,60,64] that highlighted the ability of the crop to produce different lint and seed yields when the sowing time was changed. In particular, Khan et al. [60] noted that, at early stage, crops exploited soil moisture and nutrients more efficiently over a longer growing season and produced more bolls than at late stage. This concept has been confirmed by other authors [20,64] who stated that early-sown crops were able to reap the full benefits of soil fertility during the extended growing season, and this permitted the flower bud to form and the last bolls to mature due to a sufficient accumulation of growing degree days. Therefore, as reported by Haung [64], cotton yields increase with increases in the length of the growing season based on sowing times. On the contrary, it is reasonable to suppose that, in delaying sowing, the time required by the plant to achieve full bloom and maturity tends to decrease due to higher air temperatures and warmer weather. Reductions in lint and seed yields induced by the late sowing times in our study agree with the literature [20,21,27,60]. However, despite the fact that the highest lint yields were obtained at early sowing, it is important to note that, when cotton is sown too early, a number of critical environmental factors, such as an abrupt drop in air or inadequate soil temperatures, may adversely affect optimal conditions for germination and emergence of the seedlings. In USA, in a study on the effects of different planting dates on cotton lint fiber and fiber quality [67], authors reported that analysis of air temperatures showed that early planting increased lint yield and micronaire by maximizing growing degree days; however, a high-vigor seed was required due to the fact that low soil temperatures restricted the germination process. The authors stated that adapting genetic breeding to an early sowing time strategy might include selecting for improved seed vigor and cold germination with acceptable yield and fiber quality traits. Consequently, the knowledge of some genetic characteristics of cotton seed appears fundamental when the crop is sown earlier than the normal date. In Sicily, on the basis of our findings, the most suitable sowing time for upland cotton seems to be the earliest sowing time. 

Concerning the indices of agronomic earliness, EI was of particular interest and was significantly influenced by experimental station and sowing time. In particular, the best performance of EI was obtained at normal sowing time, unlike MMD and PRI. This can be explained considering the environmental conditions which were recorded at this time. It is likely that a set of climate and soil factors, such as air temperatures, rainfall, and water and nutrient availability in soil, positively affected plant growth and yield to a greater extent than those observed at early and late dates. 

Therefore, due to favorable environmental conditions, plants grew well, and lint weight at the first harvest was found to be higher than the lint weight at successive harvests. In contrast, at early and late sowing times, fewer differences were found between lint weight recorded at the first harvest and that of successive harvests due to the fact that environmental conditions were not ideal in order to obtain high cotton lint yields.

## 5. Conclusions

The results of this study allow us to make positive considerations on the possible re-introduction of upland cotton in traditional cropping systems in semi-arid areas of Sicily. All 15 cultivars exhibited specific adaptation ability to Sicilian climate conditions and obtained good performances in terms of productivity. Cotton yield and yield components were significantly affected by environment, sowing time, cultivar and interactions between the main factors. In particular, the highest lint and seed yields were produced at the earliest sowing times. In the 1980s, in Sicily, it was found that both early and late dates were indifferently assessed the most convenient sowing times for cotton growth and yield, often determining conflicting results between various cultivation areas. However, it is worth remembering that, at the end of March or in the first 10-day period in April, low soil temperatures or a drop in air temperature could restrict germination and emergence processes in seedlings. Therefore, in order to avoid risk, the use of improved cotton cultivars for seed vigor or cold germination at early sowing times is suggested, or the planning of sowing at a time when environmental conditions are favorable for seed germination. Among the indices of agronomic earliness, the earliness index was significantly influenced by all the main factors, and this demonstrates that earliness can vary based on cultivation, environmental and genetic factors; therefore, all the factors should be considered together. Further studies are needed to investigate the effects of other agronomic practices on cotton growth and yield in Sicily in addition to the evaluation of a number of economic aspects, such as those related to the cotton fiber production chain, if Sicilian farmers were to begin growth of this crop once again. 

## Figures and Tables

**Table 1:** List of cotton cultivars in the study in 2011.

Cultivar	Species	Origin	Producer
1 J	*Gossypium hirsutum* L.	Spain	Asgrow Guadalsem
2 D	*Gossypium hirsutum* L.	Spain	Asgrow Guadalsem
3 C	*Gossypium hirsutum* L.	Spain	Asgrow Guadalsem
4 T	*Gossypium hirsutum* L.	Spain	Asgrow Guadalsem
Juncal	*Gossypium hirsutum* L.	Spain	Asgrow Guadalsem
Celia	*Gossypium hirsutum* L.	Greece	Bayer
Flora	*Gossypium hirsutum* L.	Greece	Bayer
Julia	*Gossypium hirsutum* L.	Greece	Bayer
Elsa	*Gossypium hirsutum* L.	Greece	Bayer
Claudia	*Gossypium hirsutum* L.	Greece	Bayer
5 A (hybrid)	*Gossypium barbadense* L.	Spain	Hazera Genetics
DP 419	*Gossypium hirsutum* L.	Greece	Monsanto
Alexandro	*Gossypium hirsutum* L.	Spain	Ortiz
ST 373	*Gossypium hirsutum* L.	Greece	Pioneer
ST 457	*Gossypium hirsutum* L.	Greece	Pioneer

**Table 2:** Phenological characteristics of 15 cotton cultivars in during the year 2011 and main indices of agronomic earliness used in the study.

Cultivar	Plant Density (Plant m^−2^)	Sowing-Beginning Flowering (Days)	Sowing-Beginning Boll Opening (Days)	MMD (Days)	PRI (kg ha^.1^ day^.1^)	EI (%)
Elsa	12.30 ab	76.71 ac	118.30 bc	136.20 ad	23.30 a	41.22 bd
Alexandro	10.90 ab	75.71 bd	118.00 c	134.60 d	21.82 ab	54.10 a
DP 419	10.60 ab	79.30 ab	124.00 a	137.82 ac	21.12 ab	32.64 de
Juncal	11.41 ab	73.31 de	119.01 bc	134.60 d	21.02 ab	53.20 a
2 D	12.03 ab	74.33 cd	118.00 c	135.51 cd	20.53 ab	50.41 ac
Claudia	12.90 a	78.71 ab	122.02 ac	138.60 a	20.04 ac	29.71 de
ST 373	11.31 ab	75.70 bd	123.71 a	136.91 ad	20.22 ab	39.22 ce
5 A	10.60 ab	69.00 e	122.71 ab	138.01 ac	19.81 ac	29.00 e
1 J	10.92 ab	78.02 ac	118.00 c	135.71 bd	19.04 bc	47.81 ac
4 T	11.31 ab	78.01 ac	121.31 ac	138.12 ab	18.30 bc	34.31 de
Flora	10.92 ab	80.00 a	122.31 ac	137.10 ad	18.32 bc	35.60 de
Celia	9.90 b	79.32 ab	120.71 ac	136.60 ad	18.15 bc	35.11 de
ST 457	10.31 ab	78.30 ab	123.70 a	138.33 ab	17.60 bc	33.04 de
Julia	10.62 ab	80.00 a	121.73 ac	137.71 ac	17.53 bc	30.93 de
3 C	12.00 ab	74.31 cd	119.04 bc	135.02 d	15.90 c	52.41 ab

Means followed by the same letter are not significantly different for *p* ≤ 0.05 according to Tukey test.

**Table 3:** Main morphological and yield traits of 15 cotton cultivars during the year 2011.

Cultivar	Plant Height (cm)	First Fruiting Branch Height (cm)	No. Open Boll (per Plant)	Boll Weight (g)	Lint Yield (t ha^−1^)	Lint (%)
Elsa	77.40 b	23.30 a	5.80 ad	4.47 be	1.83 a	42.20 b
Alexandro	64.10 h	14.20 b	6.31 ab	4.27 cf	1.81 a	38.21 fg
DP 419	68.71 f	16.41 ab	5.80 ad	4.74 ad	1.77 ab	38.70 fg
Juncal	73.20 c	21.80 ab	6.21 ac	3.93 ef	1.72 ab	39.31 ef
2 D	77.30 b	19.82 ab	5.42 bd	4.28 cf	1.62 ab	41.70 bc
Claudia	66.50 g	21.40 ab	4.60 d	4.73 ad	1.52 bc	44.90 a
ST 373	61.71 i	20.80 ab	5.01 cd	4.89 ac	1.64 ab	40.81 be
5 A	83.50 a	14.91 ab	6.80 a	3.84 f	1.84 a	32.70 h
1 J	71.11 de	17.71 ab	5.51 ad	4.31 cf	1.61 ab	37.31 g
4 T	72.70 cd	18.10 ab	5.52 ad	4.22 df	1.57 bc	38.02 fg
Flora	57.90 j	16.32 ab	4.80 d	4.98 ab	1.51 bc	39.71 df
Celia	61.70 i	18.03 ab	4.91 cd	5.12 a	1.49 bc	39.92 cf
ST 457	65.00 gh	15.61 ab	5.10 bd	4.66 ad	1.46 bc	39.90 cf
Julia	60.90 i	18.71 ab	4.80 d	4.78 ad	1.41 bc	41.61 bd
3 C	69.30 ef	21.90 ab	4.50 d	3.99 ef	1.35 c	36.90 g

Means followed by the same letter are not significantly different for *p* ≤ 0.05 according to Tukey test.

**Table 4:** Effect of sowing times on phenology of upland cotton cultivars during 2012.

Sowing Time	Sowing- Emergence (Days)	Sowing- Beginning Flowering (Days)	Sowing- Beginning Boll Opening (Days)	GDD_a_ (Days)	GDD_b_ (Days)	GDD_c_ (Days)
ExpSt_1						
31st March	19.51	94.83	141.16	39.51	550.91	1088.11
21st April	9.66	77.51	124.16	42.65	538.81	1091.21
12th May	16.83	71.83	117.33	86.65	630.11	1124.65
ExpSt_2						
19th April	10.83	78.01	150.33	37.81	540.55	1360.25
30th April	10.33	76.66	142.11	40.95	631.51	1357.05
14th May	9.33	81.24	128.01	31.33	786.35	1278.82

GDD_a_ = growing degree days to emergence; GDD_b_ = growing degree days to beginning of flowering; GDD_c_ = growing degree days to beginning of boll opening.

**Table 5:** Cotton plant phenology and earliness derived from different experimental stations, sowing times and cultivars during the year 2012.

	Plant Density (Plants m^−2^)	Sowing-Emergence (Days)	Sowing-Beginning Flowering (Days)	Sowing-Beginning Boll Opening (Days)	MMD (Days)	PRI (kg ha^−1^ day^−1^)	EI (%)
Experimental station (ExpSt)							
ExpSt_1	25.08 a	15.33 a	81.39 a	127.55 b	166.22 a	24.93 a	51.19 b
ExpSt_2	24.58 a	10.17 b	78.67 b	140.11 a	151.36 b	23.40 a	60.22 a
Sowing time (S)							
S_1_ (early sowing time)	24.49 a	15.17 a	86.42 a	145.75 a	157.63 b	29.84 a	50.84 b
S_2_ (normal sowing time)	24.89 a	10.00 c	77.08 b	133.08 b	157.77 b	24.74 b	67.51 a
S_3_ (late sowing time)	25.12 a	13.08 b	76.58 b	122.67 c	160.96 a	17.90 c	48.75 b
Cultivar (Cv)							
Elsa	25.22 a	12.44 a	79.44 a	133.33 a	158.65 a	25.41 a	59.38 a
Juncal	24.45 a	13.05 a	80.11 a	134.33 a	158.92 a	22.92 b	52.02 b
Source of Variance (*p*-value)							
ExpSt	0.454	0.000	0.000	0.000	0.000	0.147	0.011
S	0.732	0.000	0.000	0.000	0.000	0.000	0.000
Cv	0.253	0.155	0.727	0.274	0.356	0.022	0.034
ExpSt x S	0.426	0.000	0.000	0.001	0.000	0.440	0.000
ExpSt x Cv	0.033	0.241	0.035	0.902	0.044	0.009	0.016
S x Cv	0.748	0.165	0.613	0.574	0.000	0.094	0.002
ExpSt x S x Cv	0.745	0.281	0.269	0.022	0.000	0.101	0.000

Means followed by the same letter are not significantly different for *p* ≤ 0.05 according to Tukey test.

**Table 6:** Cotton plant growth characteristics, yield and yield components in response to different experimental stations, sowing times and cultivars during the year 2012.

Treatment	Plant Height (cm)	First Fruiting Branch Height (cm)	No. Open Bolls (per Plant)	Boll Weight (g)	Lint Yield (t ha^−1^)	Seed Yield (t ha^−1^)	Lint (%)
Experimental station							
ExpSt_1	87.93 b	36.77 b	3.98 a	3.88 b	1.74 a	2.39 a	42.17 a
ExpSt_2	103.1 a	67.42 a	4.45 a	5.03 a	1.47 b	2.06 b	41.41 b
Sowing time							
S_1_	96.38 a	53.37 a	4.53 a	4.51 a	1.99 a	2.71 a	42.29 a
S_2_	96.33 a	52.28 a	4.00 a	4.44 a	1.62 b	2.26 b	41.75 a
S_3_	93.84 a	50.65 a	4.10 a	4.41 a	1.20 c	1.70 c	41.33 a
Cultivar							
Elsa	95.52 a	52.28 a	3.99 a	4.52 a	1.72 a	2.29 a	42.80 a
Juncal	95.52 a	51.98 a	4.44 a	4.39 a	1.48 b	2.16 a	40.77 b
Source of Variance (*p*-value)							
ExpSt	0.000	0.000	0.102	0.000	0.001	0.003	0.043
S	0.605	0.336	0.250	0.770	0.000	0.000	0.104
Cv	0.996	0.876	0.104	0.282	0.001	0.183	0.000
ExpSt x S	0.002	0.012	0.013	0.000	0.106	0.374	0.066
ExpSt x Cv	0.959	0.028	0.526	0.833	0.073	0.002	0.000
S x Cv	0.770	0.000	0.058	0.359	0.114	0.300	0.259
ExpSt x S x Cv	0.039	0.000	0.012	0.615	0.291	0.771	0.139

Means followed by the same letter are not significantly different for *p* ≤ 0.05 according to Tukey test.

**Table 7:** Relationship between GDDs and cotton yields.

Sowing Time	GDDs (Days)	Lint Yield (t ha^−1^)	Seed Yield (t ha^−1^)
ExpSt_1			
31st March	1459	2.04 a	2.81 a
21st April	1416	1.75 b	2.41 b
12th May	1313	1.42 c	2.08 c
ExpSt_2			
19th April	1581	1.94 a	2.59 a
30th April	1542	1.49 b	2.11 b
14th May	1480	0.99 c	1.45 c

Means followed by the same letter are not significantly different for *p* ≤ 0.05 according to Tukey test.

## Supplementary Materials

The following are available online at https://www.mdpi.com/2223-7747/9/9/1209/s1, Figure S1: A view of “Piana di Gela Contrada Rinazzi” experimental field (ExpSt_1). Figure S2: A view of “Orleans” experimental field (ExpSt_2). Figure S3: Beginning of flowering stage at early sowing time (ExpSt_1). Figure S4: Beginning of bolls opening at normal sowing time (ExpSt_2). Table SI: Average monthly air temperature and rainfall during a multi-year average (2002–2012) in the ExpSt_1. Table S2: Average monthly air temperature and rainfall during a multi-year average (2002–2012) in the ExpA_2. Table S3: Cotton plant phenology, plant growth characteristics, yield and yield components, earliness and productivity in response to different experimental stations, sowing times and cultivars during the year 2012. Video S1: Lab saw cotton gin machine in action.
