# Supplementary material for: Effects of Environment and Sowing Time on Growth and Yield of Upland Cotton (Gossypium hirsutum L.) Cultivars in Sicily (Italy)
Source: Plants (Basel). 2020 Sep 15;9(9):1209. doi: 10.3390/plants9091209 (PMC7569890; doi:10.3390/plants9091209)
Supplement: Supplementary file 1 [file plants-09-01209-s001.zip › Tables S1 S2.docx]

**Table S1.** Average monthly air temperature and rainfall during a multi-year average (2002-2012) in the ExpSt_1.

| **Month** | **Minimum Air Temperature (°C)** | **Maximum Air Temperature (°C)** | **Rainfall (mm)** |
| --- | --- | --- | --- |
| January | 5.01 | 15.81 | 85.71 |
| February | 4.06 | 15.77 | 62.81 |
| March | 5.42 | 17.72 | 50.31 |
| April | 8.34 | 21.32 | 50.51 |
| May | 11.28 | 26.65 | 10.82 |
| June | 15.99 | 30.99 | 7.88 |
| July | 18.83 | 33.64 | 7.81 |
| August | 19.63 | 33.38 | 1.21 |
| September | 17.68 | 29.12 | 53.04 |
| October | 14.29 | 25.35 | 69.67 |
| November | 10.47 | 21.26 | 65.29 |
| December | 6.67 | 17.26 | 88.47 |

**Table S2.**  Average monthly air temperature and rainfall during a multi-year average (2002-2012) in the ExpSt_2.

| **Month** | **Minimum Air Temperature (°C)** | **Maximum Air Temperature (°C)** | **Rainfall (mm)** |
| --- | --- | --- | --- |
| January | 7.93 | 16.02 | 85.90 |
| February | 7.51 | 15.72 | 87.63 |
| March | 9.02 | 18.05 | 66.67 |
| April | 11.65 | 21.01 | 62.36 |
| May | 14.83 | 24.72 | 19.81 |
| June | 19.09 | 28.81 | 13.82 |
| July | 22.12 | 31.45 | 6.18 |
| August | 22.33 | 31.72 | 8.45 |
| September | 19.56 | 28.14 | 91.01 |
| October | 15.97 | 24.91 | 104.09 |
| November | 12.37 | 21.55 | 61.64 |
| December | 9.62 | 17.63 | 114.18 |
