# Supplementary material for: Effects of Environment and Sowing Time on Growth and Yield of Upland Cotton (Gossypium hirsutum L.) Cultivars in Sicily (Italy)
Source: Plants (Basel). 2020 Sep 15;9(9):1209. doi: 10.3390/plants9091209 (PMC7569890; doi:10.3390/plants9091209)
Supplement: Supplementary file 1 [file plants-09-01209-s001.zip › Figures S1 S2 S3 S4.docx]

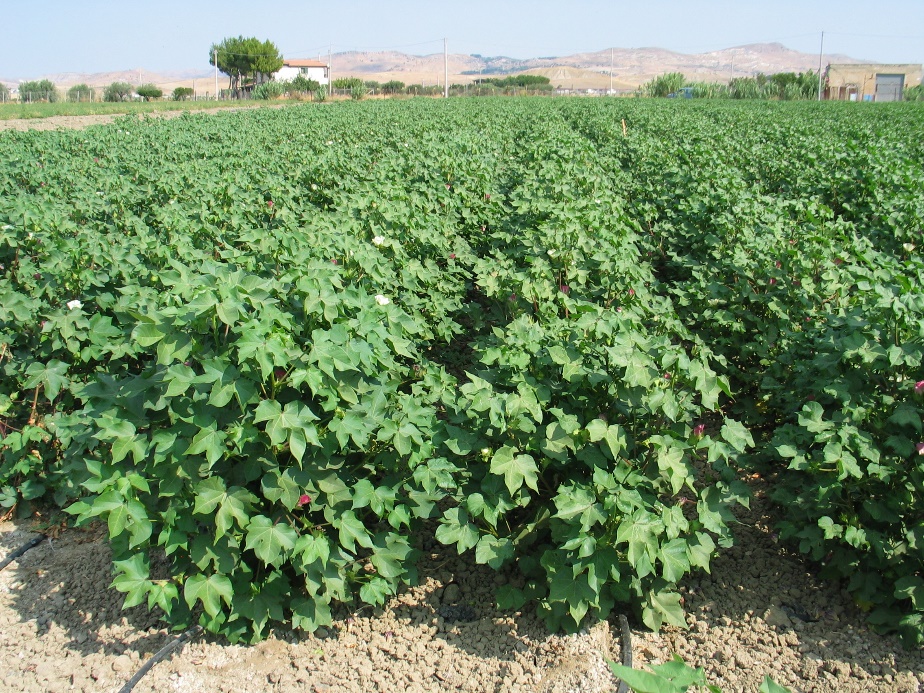


**Figure S1**. A view of “Piana di Gela Contrada Rinazzi” experimental field (ExpSt_1).


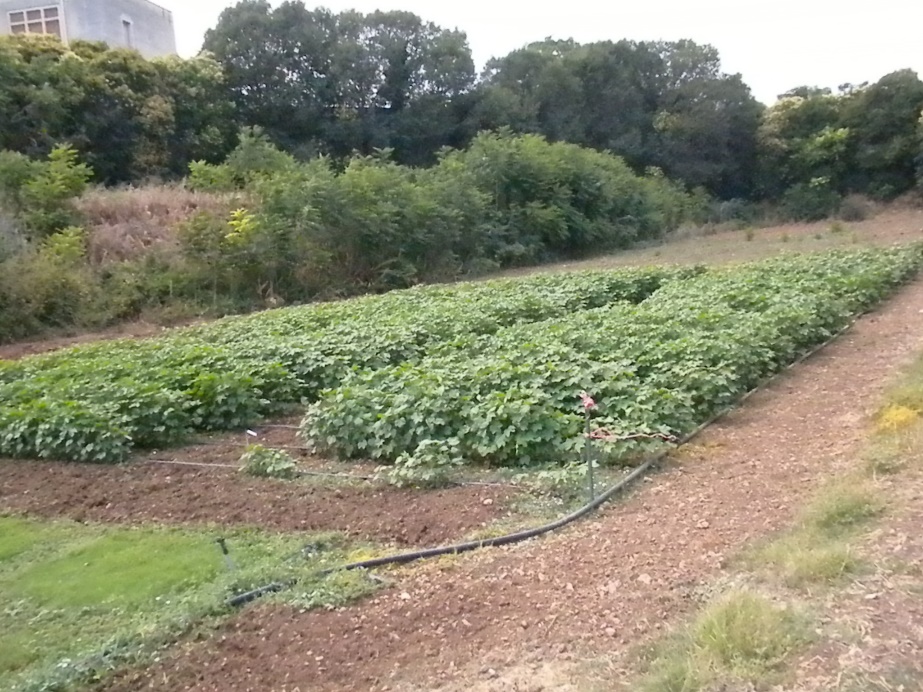


**Figure S2**. A view of “Orleans” experimental field (ExpSt_2).


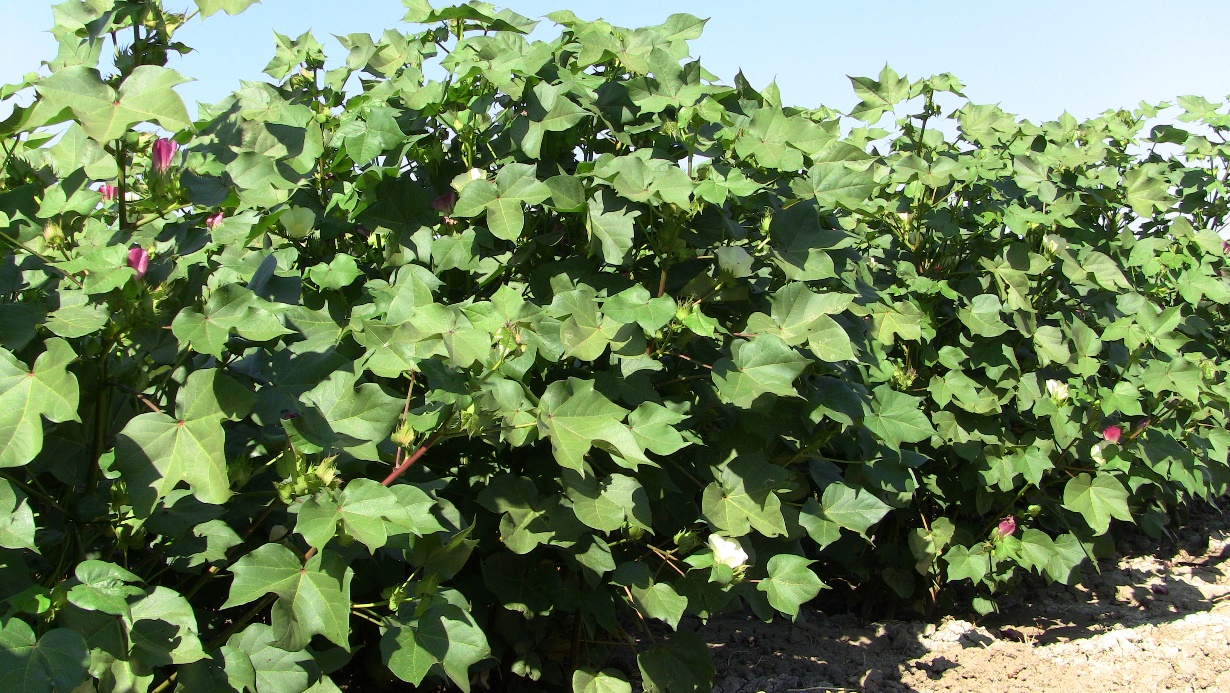


**Figure S3**. Beginning of flowering stage at early sowing time (ExpSt_1).


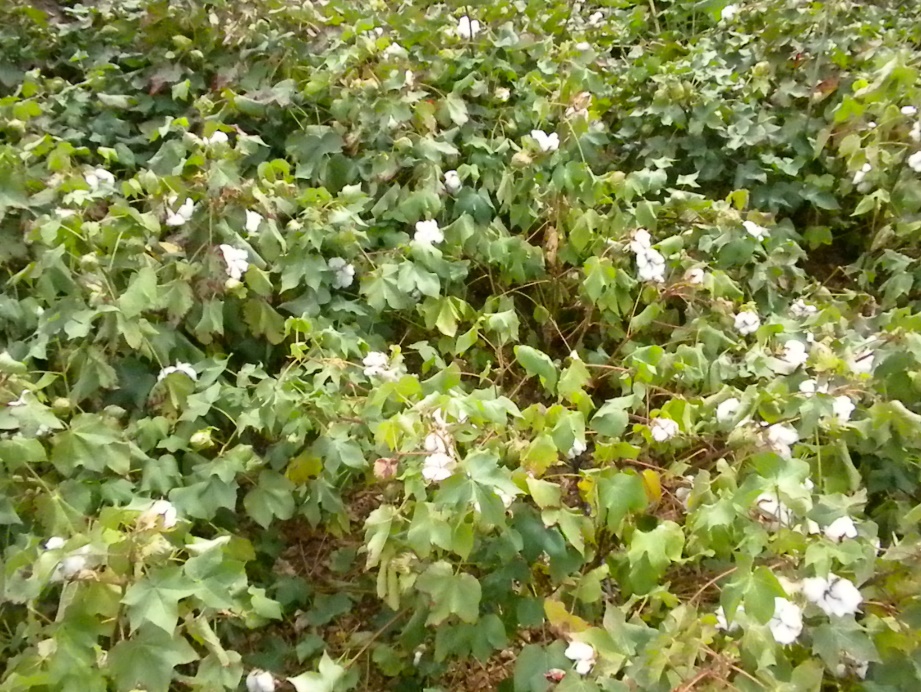


**Figura S4** Beginning of bolls opening at normal sowing time (ExpSt_2).
